# Knowledge, causes, and experience of inter-professional conflict and rivalry among healthcare professionals in Nigeria

**DOI:** 10.1186/s12913-022-07664-5

**Published:** 2022-03-09

**Authors:** Elijah N. A. Mohammed

**Affiliations:** Pharmacists Council of Nigeria, Plot 7/9 Industrial Layout, Idu, P.M.B 415 Garki, Abuja, Federal Capital Territory, Nigeria

**Keywords:** Health sector, Reform, Policy, Healthcare workers

## Abstract

**Introduction:**

The healthcare workforce is regarded as an essential component of any functioning health system, and a lack of optimal collaboration among this group can result to poor quality healthcare services to the population. In Nigerian setting, the health sector is faced with challenges of inter-professional conflict and rivalry. This study aimed at understanding knowledge, causes, and experience of inter-professional conflict and rivalry among healthcare professionals in Nigeria.

**Methods:**

A cross sectional study was undertaken to administer questionnaires to healthcare personnel in various healthcare facilities in Nigeria. Data were analysed using Statistical Package for Social Sciences.

**Results:**

A total of 2207 valid responses were received, and male participants were in majority as indicated by 63.7% of the sample. Collectively, doctors and pharmacists represented two-thirds of the sample, and majority of the participants were in the public sector (82.5%). Disparity in salary structure was the highest source of conflict. Whilst almost all the participants indicated that inter-professional rivalry and conflict are prevalent in health sector, about three-quarters of them (73.2%) disagreed that this practice is productive. A considerable number of the respondents had experienced inter-professional conflict and rivalry.

**Conclusion:**

Evidence from this study can help policymakers in developing framework that can be utilised in addressing rivalry and conflict in the healthcare sector.

## Introduction

Conflict is a challenge common to many sectors with healthcare sector inclusive [1]. In this context, conflict refers to clash of interest, opinion, or principles among healthcare workers. Whilst rivalry is associated with engaging in competitive relationship, and this can also result to conflict. Available evidence suggests that conflict is significantly higher in the health sector due multifaceted and regular interaction among healthcare professionals [2]. Conflict is a phenomenon that can arise at all levels within the healthcare space and has been identified as a global problem [3–7]. The primary obligation of healthcare workers is the well-being of patients [8]. However, factors such as organisation hierarchy, specialisation, and multiplicity of skills have created rivalry among this group of service providers [9]. Another notable factor, which has contributed to rivalry is power struggle among various groups of healthcare professionals over the control and leadership of work process [10]. Also, various health professional associations act as interest groups to influence government policy in favour of their members, ignoring the implication to other professional groups and the health sector in general.

Provision of healthcare services involves multidisciplinary measure which requires doctors, pharmacists, nurses, medical laboratory scientists and other healthcare professionals with different area of specialisation to work as a team [11]. Multidisciplinary collaboration among healthcare workers can optimise quality of care and result in improved outcome for patients [12].

Inter-professional rivalry is detrimental to both patients and the healthcare system. Available evidence suggests that conflict or rivalry in the health sector disrupts intra and inter sectorial collaboration [13], and can also result to aggravated stress including emotional exhaustion for healthcare workers. Inter-professional rivalry has also been implicated in reducing the commitment of healthcare workers to healthcare services [14], as well as encouraging selfish behaviour [15], which consequently results in mistreatment or non-treatment of patients.

The Nigerian health sector has witnessed unhealthy rivalry among healthcare professionals for decades. This has led to several industrial action such as strike [10], and thereby hampering sustainable development in the sector. The rivalry in the sector is mostly between medical doctors and other healthcare professionals [16]. Whilst other healthcare professionals have blamed doctors of being at the helm of affairs of the entire sector, the doctors on their part had also alleged other healthcare professionals of uniting together to fortify a joint force against them [17]. The healthcare delivery system in Nigeria is a blend of private and the three tiers of government with Federal Government responsible for the affairs of tertiary health facilities, the State Governments taking charge of secondary health facilities, and the Local Governments in charge of the affairs of primary healthcare centres [18]. Available evidence reveals the existence of incessant rancour among healthcare professionals in the country, with a number of such rancour degenerating into full-blown conflict and sometimes industrial dispute [10]. Despite the vulnerability of the Nigerian health sector to conflict situations, only few studies have been undertaken in this area. It is against this backdrop that this study therefore aimed at understanding knowledge, causes, and experience of inter-professional conflict and rivalry among healthcare professionals in Nigeria.

## Methods

A cross-sectional survey was undertaken in Nigeria between April and June 2021. A data collection tool was designed in English language following an extensive literature review [3, 4, 6, 16, 17]. An iterative process involving a panel of faculty members in health sciences was used to develop the items. The panel consisted of 4 members from two institutions. Members of the panel were engaged in teaching and research activities in this area. A draft version of all the items in the instrument was reviewed by the panel; each person reviewed the items independently and suggested changes, additions, and deletions. The revision process continued until a consensus was reached. The questionnaire items were structured to gain insights on knowledge, causes, and experience of inter-professional conflict and rivalry among healthcare professionals in Nigeria.

Face and content validation of the instrument were undertaken using an independent expert panel comprising 6 faculty members. The questionnaire was assessed for appropriateness, complexity, attractiveness and relevance. Some of the statements were edited and reworded, whilst content validity was evaluated by quantitative method. Content validity ratio and content validity index were tested for each item, and only those that passed these tests were included in the final questionnaire. The questionnaire was pretested by administering it to initial 30 participants comprising different healthcare profession selected randomly, the feedback received did not warrant any major change. Data were collected using online and physical methods of questionnaire administration, this was to ensure that a good number of healthcare professionals participated in the study. The participants were selected using stratified sampling method. Two states were selected randomly from six geopolitical regions in Nigeria. Snowball sampling strategy was employed during the online data collection process [19, 20], and this involved the use of WhatsApp. The link to the survey was sent to various groups made up of current practicing healthcare professionals, participants were asked to send the questionnaire to their colleagues practicing in the same state with them. Hard copies of questionnaires were administered physically to doctors, pharmacists, medical laboratory scientists, nurses, and other healthcare workers in several healthcare facilities that were randomly selected from each state using convenience sampling method. The physical administration commenced after the link to online data collection was closed. Prior to the paper-based questionnaire administration, participants were requested to indicate if they had previously responded to online version of the questionnaire and only healthcare practitioners who did not participate during the online data collection process were given hard copies of questionnaires to complete.

Inclusion criterial were healthcare professionals who are currently practicing in Nigeria, and with current annual license to practice. And all participants who did not meet these criterial were excluded from the study.

The study was a national survey, and data were collected from multiple health facilities in Nigeria. Ethical approval was obtained from National Health Research Ethics Committee of Nigeria, as they are responsible for granting approval for studies involving multiple centres. This approval was obtained before the commencement of data collection. Participation in the study was voluntary as informed consent was obtained prior to the administration of questionnaires. Confidentiality was maintained by not including the names of the study participants in the data collection tool.

Following the importation of data collected into Statistical Package for Social Sciences software version 25, descriptive statistical analysis was carried out. Associations between variables were tested using chi square test. A *p-*value of 0.05 or less was considered the threshold for statistical significance.

## Results

### Demography and response rate

A total of 2000 copies of paper-based questionnaires were administered, and 2,207 valid responses were received comprising 503 online and 1703 paper-based respondents. Response rate for paper questionnaire was 85.2%. Male participants represented two-thirds of the respondents. Respondents above 60 years of age were in minority, whilst those between 41 and 50 represented the most populous proportion of the sample as indicated by 32.8%. Majority of the study participants (54.1%) had first degree as their qualification. Further details about socio-demographic characteristics are presented in Table 1 below.

**Table 1:** Socio-demographic characteristics

Variable	Frequency (%)
Gender	
Male	1405 (63.7)
Female	802 (36.3)
Age	
18 – 30	309 (14.1)
31 – 40	677 (30.8)
41 -50	717 (32.8)
51 – 60	461 (21.0)
Above 60	34 (1.5)
Highest Educational Level	
Diploma	57 (2.6)
First degree	1172 (54.1)
Master’s degree	743 (34.3)
Doctorate	195 (9.0)
Profession	
Doctor	740 (33.9)
Pharmacist	687 (31.5)
Medical Laboratory Scientist	245 (11.2)
Nurse	443 (20.3)
Other healthcare workers	67 (3.1)
Grade Level	
Senior	1440 (68.2)
Directorate	356 (16.9)
Management	259 (12.3)
Others	55 (2.6)
Level of Union Activity	
No interest in union	420 (20.2)
Low activity	684 (32.9)
Average activity	712 (34.3)
High activity	260 (12.5)
Sector	
Public sector	1821 (82.5)
Private sector	219 (10.4)
Development sector	9 (0.4)
Academic sector	66 (3.1)

### Sources of conflict

**Fig. 1:**
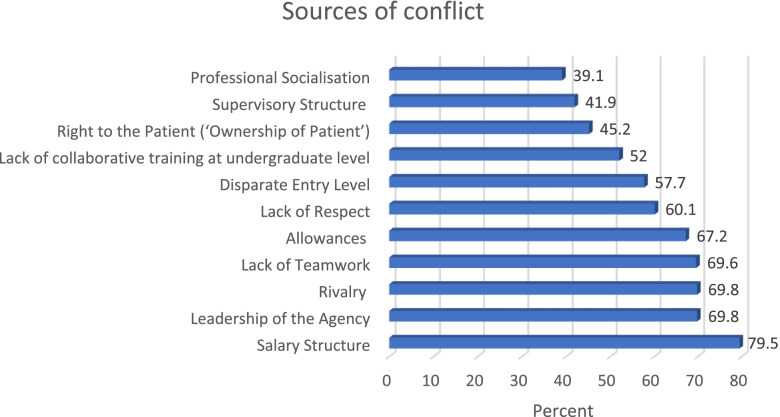
Sources of conflict in Nigerian healthcare sector

Inter-professional conflict among healthcare team is known to hinder high quality healthcare delivery. Findings in Fig. 1 shows that the most common source of conflict is salary structure as indicated by 79.5% of the study participants, and this is closely followed by leadership of agencies (69.8%) and rivalry (69.8%), whilst the least source of conflict is professional socialisation (39.1%). Lack of team work, allowances, lack of respect, disparate entry level, and lack of collaborative training at undergraduate level also have significant role in promoting conflict in the healthcare sector, as more than half of the study participants indicated each of them as sources of conflict in the sector.

### Knowledge of conflict and rivalry

**Fig. 2:**
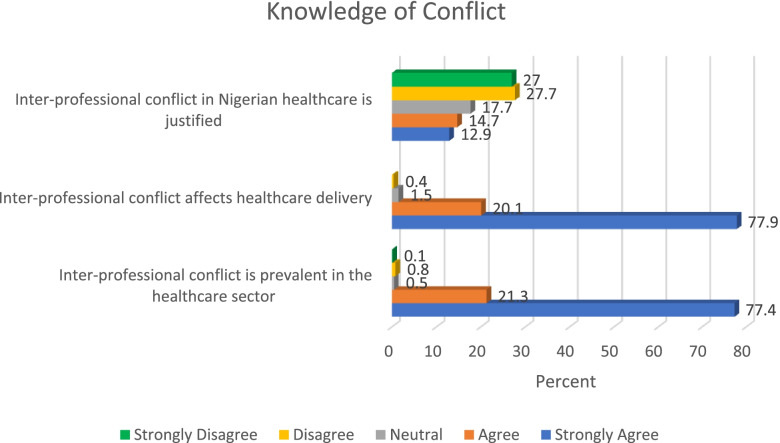
Knowledge of conflict

Findings from this study revealed that more than half of the study participants (54.7%) disagreed that inter-professional conflict in Nigerian healthcare is justified. Almost all the respondents (98%) indicated that inter-professional conflict affects healthcare delivery, whilst a similar proportion (98.7%) had also indicated that inter-professional conflict is prevalent in the health sector. Further details relating to knowledge of inter-professional conflict are presented in Fig. 2. Findings in this study shows that inter-professional conflict places patients at a disadvantaged position as a result of its effect in delivery of healthcare services. This can consequently deny patients access to high quality healthcare services.

**Fig. 3:**
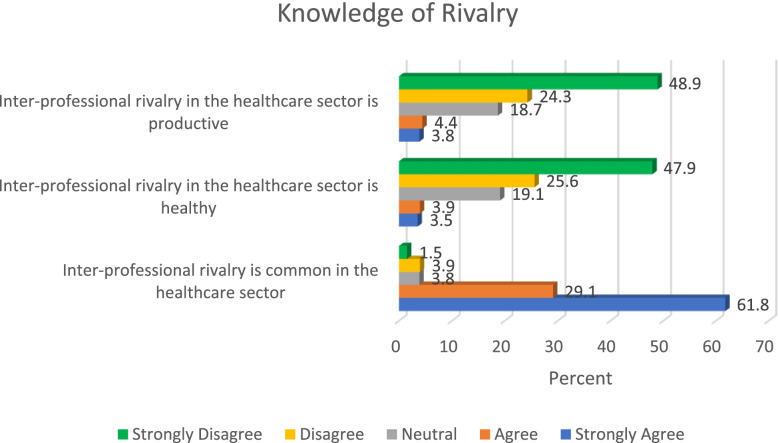
Knowledge of rivalry

Only few participants (8.2%) indicated that inter-professional rivalry in healthcare sector is productive, and a strong majority of the study participants (90.9%) indicated that inter-professional rivalry is common in the health sector. However, slightly above a quarter of the respondents (27.6%) agreed that inter-professional rivalry is justified. Other relevant details about knowledge of conflict in the healthcare sector are presented in Fig. 3. Findings in Fig. 3 also indicate that a populous proportion of the sample had good knowledge of the negative effect of inter-professional rivalry, as they clearly indicated that inter-professional rivalry is unhealthy and unproductive.

### Experience of conflict and rivalry

**Fig. 4:**
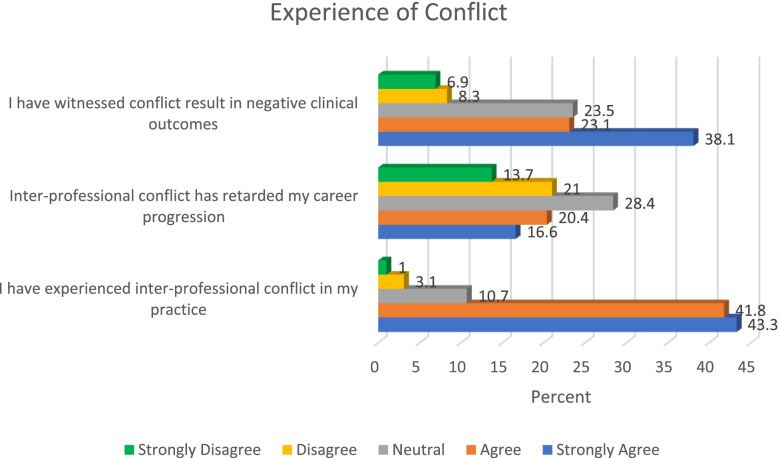
Experience of conflict

A strong majority of the study participants (85.1%) had experienced inter-professional conflict in their practice, more than a third of the respondents (37%) indicated that inter-professional conflict has retarded their career progression, and about a two-thirds of the sample (61.2%) indicated witnessing conflict resulting in negative clinical outcome. Figure 4 provides details of experience of conflict. Findings from this study therefore suggests high rate of inter-professional conflict among healthcare professionals in Nigeria. There is need to holistically address the various causes of conflict in order to achieve a peaceful milieu in Nigerian healthcare system. This is important for the purpose of patients who may be adversely affected by the impact of conflict in the sector.

**Fig. 5:**
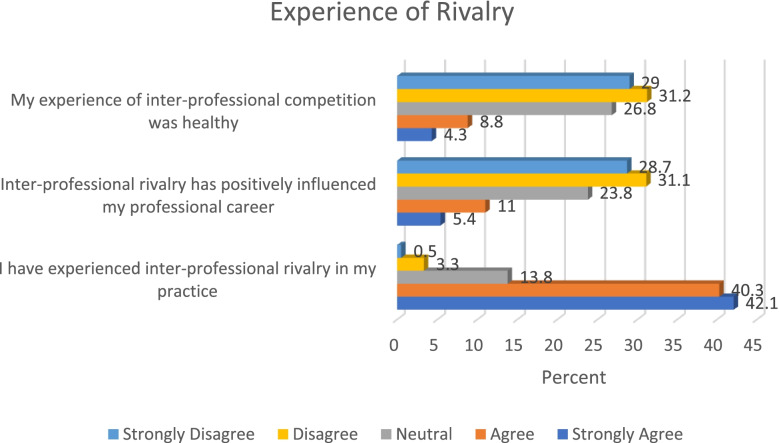
Experience of rivalry

A considerable proportion of the study participants (82.4%) indicated that they had experienced inter-professional rivalry in their practice, whilst only few participants (16.4%) agreed that inter-professional rivalry has positively influenced their career. Figure 5 below gives an overview of the study participants experience on inter-professional rivalry. In this study, findings therefore suggest that inter-professional rivalry is prevalent among healthcare professionals in Nigeria. Addressing the causes of inter-professional rivalry is critical in order to prevent conflict and crises in Nigerian healthcare sector.

Further to the descriptive statistical analysis undertaken, chi square test was carried out to determine association between variables. Findings indicate that a strong majority of doctors (84.5%), pharmacists (84.9%), medical laboratory scientists (89.5%), and nurses (77.9%) agreed that they had experienced inter-professional conflict in their practice as compared to only 70.9% of other healthcare workers not in the above categories. This finding was statistically significant (*p* < 0.001). Similarly, more doctors (78.9%), pharmacists (83.9%), medical laboratory scientists (81.5%), and nurses (87.5%) indicated that they had experienced inter-professional rivalry in their practice, compared to 74% of other healthcare workers that indicated that they had experienced inter-professional rivalry. Again, this finding was statistically significant (*p* < 0.001). Also, inter-professional rivalry seems to be common in the public sector and least prevalent in development sector as 85.6% of participants in public sector indicated that they had experienced inter-professional rivalry, which was higher than 77.8% that indicated they had experienced inter-professional rivalry in development sector. This finding too was statistically significant (*p* < 0.001).

## Discussion

Findings from this study revealed that salary structure was the most indicated source of conflict in the Nigerian healthcare sector, suggesting that salary structure has played significant role in inter-professional conflict among healthcare personnel in the country. This finding therefore suggests the need for proper harmonisation of salary component among healthcare professionals. In Nigeria, two salary scale exists for healthcare workers, with wide disparity between the salary structures [21]. The salary systems include Consolidated Medical Salary Structure (CONMESS) for physicians and dentists, and the Consolidated Health Salary Structure (CONHESS) for all other healthcare professionals. Proper review and harmonisation of these two salary structures can reduce conflict arising as result of disparity in salary, as well as prevent frequency of strike actions in the healthcare sector.

Slightly above two-thirds of the study participants attributed leadership of agencies and rivalry as sources of conflict in the healthcare sector, thereby corroborating previous study that reported these two components as some of the most common causes of strike in the healthcare sector [22]. Another source of conflict that closely followed was lack of team work, and this is comparable to previous studies that reported lack of collaboration among Nigerian healthcare workers, unlike what is obtainable in developed countries [23–25]. The implication of this finding is that patients may not receive high quality healthcare services, thereby reducing treatment outcomes. Adequate collaboration among healthcare professionals can increase quality of care. Working together as a team reduces medical errors and increases patient safety [26]. In Nigeria, healthcare workers belonging to different groups engage in fight for supremacy, the crises that erupt from this process consequently prevents optimal healthcare delivery to Nigerians [27]. In addition, teamwork reduces workloads, increases job satisfaction, improves patient satisfaction and reduces morbidity [28]. Furthermore, findings revealed that lack of respect, disparate entry level, and lack of collaborative training at undergraduate level also have considerable effect on causes of conflict among healthcare professionals. Disparity exists between the entry point of physicians and other healthcare workers, whilst healthcare professionals like pharmacists and medical laboratory scientists enters public service at salary grade level 10, the physicians are placed on entry point of salary grade level 12, and this is different from what was obtainable 3 decades ago [29].

Almost all the respondents in this study indicated that inter-professional conflict and inter-professional rivalry are common in the health sector, whilst also accepting that these practices affects healthcare delivery. These findings indicate that attaining universal health coverage in Nigeria may take longer time if contextual strategies are not developed to mitigate conflict in the health sector, as available evidence suggests that crises within the healthcare workforce is a major constraint towards global health system development and sustenance [30–32]. Although, majority of the study participants indicated that there is no justification for inter-professional conflict in the health sector, up to a quarter of the study participants indicated that inter-professional conflict in the health sector is justified, which is a source for concern. Further studies can be undertaken in this area to understand reasons for this justification. About three-quarters of the study participants were aware that inter-professional rivalry is not healthy, whilst a similar proportion also indicated that such rivalry is not also productive. These findings suggest that the participants understood the negative impact of inter-professional conflict and rivalry in the sector.

In this study, findings revealed that a strong majority of the study participants had experienced inter-professional conflict and rivalry, thereby validating previous study that reported high rate of inter-professional conflict in the health sector [32]. Furthermore, doctors, pharmacists, nurses, and medical laboratory scientists were more likely to experience inter-professional conflict and rivalry compared to other healthcare workers. Therefore, policy development to address inter-professional conflict and rivalry should be targeted more at these group of professionals so as to achieve the primary obligation of healthcare professionals, which is the well-being of patients [8, 12]. Also, findings from this study identified public sector as the sector where inter-professional conflict is more likely to be experienced, suggesting the need for desperate and urgent reforms to mitigate conflict in this area.

Regarding effect of conflict on career, findings from this study indicate that inter-professional conflict had retarded the career of more than a third of the study participants whilst only a few respondents seem to have benefited from inter-professional conflict. This also buttress the importance and need to initiate policy reforms that will promote harmonious relationship among healthcare workers. Successfully achieving this will expedite the development of Nigerian healthcare sector to international standard, and healthcare personnel in Nigeria will be more dedicated to their services [14, 33]. Interestingly, majority of the study participants were in consensus that they had witness conflict results in negative clinical outcomes, which further validates the negative effects of healthcare conflict on patients.

## Conclusion

This study has revealed a number of insightful findings regarding inter-professional rivalry and conflict in Nigerian healthcare sector. There is no better time than now to articulate strategies in mitigating and preventing rivalry and conflict in the Nigerian healthcare sector.

Inter-professional conflict and rivalry may not only hinder Nigerians’ access to high quality healthcare services, but can also result in a wastage of resources, for both the healthcare system, and patients.

Whilst this study provides evidence that validates existence of inter-professional conflict and rivalry in the healthcare sector, other findings that emerged represent an opportunity to develop contextual strategies that can appropriately address rivalry and conflict among healthcare professionals. Therefore, further studies in this area can be focused on articulating robust and comprehensive strategies in preventing rivalry and conflict in the sector.

## Data Availability

The datasets generated and analysed during this study are available on request.
